# Antibacterial Activity of Modified Sesbania Gum Composite Film and Its Preservation Effect on Wampee Fruit (*Clausena lansium* (Lour.) Skeels)

**DOI:** 10.3390/foods13050639

**Published:** 2024-02-20

**Authors:** Mingyan Wang, Dongfen Huang, Yue Sun, Guanglong Yao, Hengfu Huan, Jian Chen

**Affiliations:** 1 Tropical Crops Genetic Resources Institute, Chinese Academy of Tropical Agricultural Sciences (CATAS)/Key Laboratory of Crop Gene Resources and Germplasm Enhancement in Southern China, Ministry of Agriculture and Rural Affairs, Haikou 571101, China; wmy19166@163.com (M.W.); dongfen.huang@163.com (D.H.); sunyue327724@163.com (Y.S.); 2 Key Laboratory of Food Nutrition and Functional Food of Hainan Province, Engineering Research Center of Utilization of Tropical Polysaccharide Resources, Ministry of Education, College of Food Science and Technology, Hainan University, Haikou 570228, China; yaoguanglong@126.com

**Keywords:** sesbania gum, oxidation modification, composite film, antibacterial activity, fruit preservation

## Abstract

The primary challenges in fruit and vegetable preservation include extending storage duration while preserving sensory quality and nutritional value. In this study, sesbania gum (SG) was oxidized to prepare oxidized sesbania gum (OSG). An OSG/ZnO composite film was subsequently prepared, combining OSG, sodium carboxymethyl cellulose (CMC), and nano-zinc oxide (nano-ZnO). The preparation technology was determined via a response surface optimization experiment. When the addition amount of nano-ZnO exceeded 0.3 mg/mL, the composite films exhibited an antibacterial rate of over 90% against *E. coli* and S. *aureus*. For wampee (*Clausena lansium* (Lour.) Skeels) preservation, a OSG/ZnO-0.3 film was directly applied as a coating. The findings demonstrated favorable results in terms of the rate of rotting, soluble solids, and titrable acidity, effectively prolonging wampee fruit storage. This suggests the potential of an OSG composite film with nano-ZnO as a promising fruit packaging material, thereby expanding the application of SG and wampee fruit preservation.

## 1. Introduction

Fruits and vegetables are nutrient-rich and therefore are essential for humans. However, they easily rot after picking because of respiration, transpiration, damage caused by reactive oxygen species, and microbial pollution [1,2]. Therefore, an environmentally friendly, simple, and efficient fruits and vegetables packaging material with multiple protective functions must be developed to extend their shelf life and thus reduce food waste [3]. Because of the increase in awareness of food safety and environmental pollution, some biodegradable natural polymers have received considerable research attention for their potential use in food packaging [4,5].

Sesbania is an important green manure crop. Sesbania gum (SG) is a plant gum composed of galactomannan, with the main chain linked by the β-(1→4) glucoside bond. SG belongs to the natural galactomannan gum polymer [6,7]. However, SG has a high molecular weight, water insoluble content, and viscosity, and thus its applications are limited [8]. It is oxidized and degraded in the presence of an oxidant to produce oxidized sesbania gum (OSG). Compared with SG, OSG has a low viscosity, high transparency, and fluidity [9]. Therefore, it can be better mixed with other materials to prepare composite films. Sodium carboxymethyl cellulose (CMC) is a water-soluble cellulose derivative [10]. Because of its non-toxicity, easy water solubility, biocompatibility, and other advantages, CMC serves as a good polymer film-forming material [11,12,13]. The CMC-based film has good tensile strength. As a reinforcing agent, CMC can increase the tensile strength of the composite film [13]. The film prepared using a single polymer has many shortcomings, and the performance of such films can be improved by combining the polymer with other materials [14,15]. Nano-zinc oxide (Nano-ZnO), a type of nanomaterial, has good physical and chemical properties. Nano-ZnO-modified packaging materials exhibit improved mechanical properties and special antibacterial functions [16,17,18].

Wampee (*Clausena lansium* (Lour.) Skeels) is a genus of Rutaceae. It produces typical tropical and subtropical fruit, widely cultivated in Guangdong, Guangxi, Hainan, Fujian, and other provinces of China [19]. The fruit pulp is rich in nutrients, such as vitamins and amino acids, and functional substances such as polysaccharides and flavonoids [20]. The flesh of the wampee fruit is firm, tender, and smooth. The fruit has a sweet and sour taste and a unique and rich flavor. It is beneficial for digestion, stomach strengthening, fluid production, and thirst quenching [21]. During postharvest storage and transportation, the fruit may be mechanically damaged, which may accelerate the infection of microbial pathogens and promote fruit senescence. Therefore, developing new fresh-keeping packaging materials is particularly crucial [21,22]. Contemporary research on postharvest wampee fruit preservation predominantly includes hot water treatments and low temperature conditioning [23], irradiation preservation [24], ethanol fumigation [21], coating preservation [25], as well as the utilization of oxyresveratrol [26] and other natural active preservation agents. In this study, the non-toxic, cost-effective SG was employed as a raw material and oxidatively modified and combined with other substances to create a film with antibacterial properties for wampee fruit preservation.

SG shares several physicochemical properties with starch, which is widely studied for composite film preparation and its application in fruits and vegetables preservation. However, reports on SG film formation are scarce. To date, no research has explored the preparation of modified SG composite film and its application in fruit preservation. In this study, SG underwent modification with H_2_O_2_ as an oxidant, resulting in OSG. The composite film was prepared via the casting method by mixing OSG with glycerol and CMC. The effects of different mixed materials on the basic properties of films were determined. Antibacterial composite films were prepared using nano-ZnO as an antibacterial agent. The antibacterial film was used for wampee fruit preservation. The weight loss rate, rate of rotting, total soluble solid (TSS), and titratable acid (TA) content were measured to evaluate the preservation effect of the composite film on the wampee fruit. As a natural polymer material, SG is widely available and inexpensive. Compared with the existing film, an SG-modified composite film has easy decomposition and antibacterial activity. These findings provide ideas for exploring the application of natural bioedible modified SG films for preserving wampee fruit.

## 2. Materials and Methods

### 2.1. Materials

Sesbania gum (SG) was purchased from Xi’an Longmao Biotechnology Co., Ltd., Xi’an, China. Sodium carboxymethyl cellulose (CMC) was purchased from Shanghai Changguang Enterprise Development Co., Ltd., Shanghai, China. Nano-zinc oxide (nano-ZnO, particle size 30 nm) was purchased from Kuer Chemical Technology Co., Ltd., Beijing, China. Glycerol (56-81-5, AR), sodium hydroxide (1310-73-2, AR), sodium chloride (7647-14-5, AR), hydrochloric acid (7647-01-0, 38%), and anhydrous ethanol (64-17-5, 95%) were purchased from Xilong Scientific Co., Ltd., Guangzhou, China. The manufacturer of PE fresh-keeping film was MIAOJIE. LB culture medium was purchased from Guangdong Huankai Microbial Technology Co., Ltd., Guangzhou, China. AGAR powder was purchased from Beijing Solarbio Science & Technology Co., Ltd., Beijing, China. *Escherichia coli* and *Staphylococcus aureus* were purchased from the Guangdong Microbial Culture Collection Center, Guangzhou, China. Wampee fruits (*Clausena lansium* (Lour.) Skeels) were purchased from the orchards of local farmers in Yongxing Town, Haikou City.

### 2.2. Preparation of Oxidized SG

OSG was prepared with H_2_O_2_ as the oxidant and CuSO_4_ as the catalyst [9,27].

### 2.3. Preparation of OSG Composite Film

The OSG composite films were prepared using the solution casting method. The same amount of film-forming solution was poured into a 120 mm petri dish. When the film-forming solution did not flow, it was placed in a drying oven at 50 °C for 24 h. The film was thoroughly cooled to make it soft and removed for use [27,28,29]. Nano-ZnO of 0.1, 0.2, 0.3, 0.4 and 0.5 mg/mL were added to the composite film and were named OSG/ZnO-0.1, OSG/ZnO-0.2, OSG/ZnO-0.3, OSG/ZnO-0.4 and OSG/ZnO-0.5, respectively [30].

### 2.4. Determination of Thickness

Five points were selected on average on the composite film, and the thickness was measured using a vernier caliper. The average (unit: mm) was taken to determine the thickness of the film sample, and the mechanical and physical properties of the film sample were calculated [31].

### 2.5. Determination of Mechanical Properties

According to the method of He et al. [32] and Yuan et al. [31], the determination was performed using the TA.XT Plus texture analyzer.

### 2.6. Determination of WATER Vapor Permeability (WVP)

According to the method of Chen et al. [33] and Dong et al. [34], the composite film was tightly covered on the weighing bottle containing CaCl_2_, and WVP was calculated according to the mass difference before and after measurement [27].

### 2.7. Calculation of Comprehensive Score

The TS, EBA, and WVP of the film have a significant influence on the film preparation process. Therefore, using a single index as an evaluation index is unreasonable. This study used the information entropy weighting method to determine index weights and calculate a comprehensive score. The response surface optimization was conducted using the comprehensive score as the evaluation index. The three indexes were initially standardized within a range using Formulas (1) and (2) to mitigate the influence of different index dimensions before the analysis. TS and EAB were positive indicators, whereas WVP was a negative indicator [35].

(1) $$Positive\;indicator\;p=\frac{X_{i}-X_{min}}{X_{max}-X_{min}}$$

(2) $$Negative\;indicator\;p=\frac{X_{max}-X_{i}}{X_{max}-X_{min}}$$

p indicates the standardized value; $X_{i}$ indicates the value to be analyzed; and $X_{max}$/$X_{min}$ indicate the maximum value and minimum value of the column where the value to be analyzed resides.

The weight coefficients of TS, EAB and WVP are 0.483, 0.218 and 0.299, respectively. The comprehensive score = TS × 0.483 + EAB × 0.218 + WVP × 0.299.

### 2.8. Antibacterial Activity

The antibacterial activity of the OSG/ZnO composite film against two common pathogenic bacteria, *E. coli* and *S. aureus*, was studied using the antibacterial rate method.

Strain activation: For activation, 2% inoculum of each *E. coli* and *S. aureus* was inoculated separately into liquid LB medium and cultured in a 37 °C incubator for 24 h. This operation was repeated to activate two generations.

Antibacterial rate test: The activated bacterial solution was diluted into a bacterial suspension at approximately 1.0 × 10^−6^ CFU/mL. Then, 0.01 g of the composite membrane was accurately weighed, immersed in 5 mL bacterial suspension, placed in an oscillating incubator at 37 °C, and cultured for 3 h at a rotating speed of 150 rpm/min. Following culture, 100 μL of the sample solution was evenly coated on the LB agar plate. The plate was placed upside down in a constant temperature incubator at 37 °C for 24 h, and the number of colonies was recorded [30,36].

The antibacterial rate was calculated as follows (3):

(3) $$Antibacterial\;rate(\%)=\frac{A-B}{A}\times 100\%$$

where A is the number of colonies of oxidized sesbania gum as the control, and B is the number of colonies containing the nano-ZnO composite film.

### 2.9. Application of Composite Films in Wampee Fruit Preservation

#### 2.9.1. Wampee Fruit Processing

For the experiments, we used wampee fruits with no obvious scars on the surface. All fruits were uniform and achieved consistent maturity. The experiment included five treatment groups: blank, PE film coating, OSG/CMC film coating, OSG/ZnO-0.3 film coating, and OSG/ZnO-0.3 film liquid coating. Three parallels were maintained for each group, with each parallel group containing 6 selected wampee fruits. In the film coating group, the wampee fruits were closely coated with the prepared composite film. In the film liquid coating group, the experimental wampee fruits were immersed in the OSG/ZnO-0.3 composite film liquid for 2 min, removed, and dried in a ventilated place. The quality index of wampee fruits were determined after every two days of storage [36,37].

#### 2.9.2. Weight Loss Rate

The calculation was made by using the weighing method [33]. Briefly, the weight of each group was measured every two days of storage. The weight loss rate was calculated in accordance with Formula (4):

(4) $$Weight\;loss\;rate(\%)=\frac{m_{0}-m}{m_{0}}\times 100\%$$

where m_0_ is the initial mass (g) of the wampee fruit, and m is the mass (g) of the wampee fruit after storage for a certain number of days.

#### 2.9.3. Rate of Rotting

The observation method was used to observe the rotting of the wampee fruit, and fruits with black spots, brown spots, and mold were all identified as rotten fruits [30]. The rotting rate was calculated according to Formula (5):

(5) $$\mathrm{Rate}\;\mathrm{of}\;\mathrm{rotting}(\%)=\frac{\mathrm{number}\;\mathrm{of}\;\mathrm{rotten}\;\mathrm{fruits}}{\mathrm{Initial}\;\mathrm{total}\;\mathrm{number}\;\mathrm{of}\;\mathrm{fruits}}\times 100\%$$

#### 2.9.4. Determination of Total Soluble Solid Content

Drops of wampee fruit juice were placed on the glass surface of a hand-held sugar meter to determine the TSS content [4].

#### 2.9.5. Determination of Titrable Acid

TA was determined using a previous titration method [38], with a slight modification. First, 2 g of ground pulp was weighed and added into a 10 mL centrifuge tube, and 5 mL of distilled water was added to the same tube. The solution was centrifuged at 4 °C and 10,000 rpm/min for 10 min. The supernatant was collected in a 50 mL volumeter bottle and shaken well for future use. Then, 10 mL of constant volume liquid was added to a 50 mL conical bottle, followed by the addition of 2 drops of phenolphthalein. This mixture was titrated with 0.01 mol/L NaOH. The dosage was recorded when the solution turned slightly red and did not fade for 30 s. This procedure was repeated three times to determine the average, and TA was calculated as follows (6):

(6) $$TA(\%)=\frac{C\times V_{1}\times K\times B\times 100}{V_{0}\times W}$$

where C is the NaOH concentration (0.01 mol/L), V_1_ is the NaOH dosage (mL), V_0_ denotes the volume of the liquid sample absorbed during titration (mL), B represents the total volume of the liquid sample at a constant volume (mL), W denotes the sample quality (g), and K is the acidity conversion coefficient. Moreover, 0.064 is the conversion factor for citric acid (K = 0.064).

### 2.10. Statistical Analysis

The data in this experiment were the mean of 3 replicates. The results are expressed as mean ± standard deviation. SPSS 26 software and origin 2021 software were used for statistical analysis and mapping.

## 3. Results and discussion

### 3.1. Effects of OSG, CMC, and Glycerol Additions on TS, EAB and WVP of Composite Films

**Figure 1:**
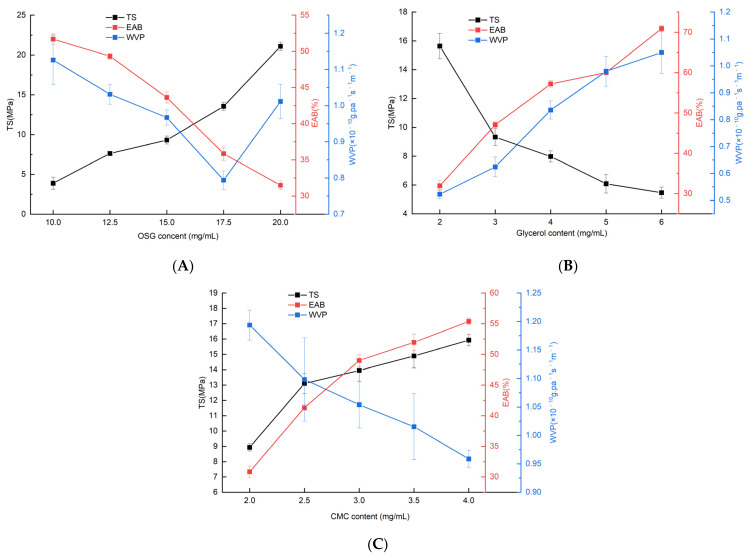
Effect of OSG, glycerol, CMC content on TA, EAB, WVP of composite films. (**A**) Reaction conditions: amount of glycerol: 3 mg/mL; amount of CMC: 3 mg/mL. (**B**) Reaction conditions: amount of OSG: 15 mg/mL; amount of CMC: 3 mg/mL. (**C**) Reaction conditions: amount of OSG: 15 mg/mL; amount of glycerol: 3 mg/mL.

Figure 1A–C exhibit the effects of OSG, glycerol, and CMC contents on the TS, EAB and WVP of composite films, respectively. When the OSG supplemental level was 10–20 mg/mL (Figure 1A), the TS of the composite film tended to increase with an increase in the OSG content, whereas EAB decreased significantly. This indicates that the rigidity of the composite film increased, whereas its toughness gradually decreased. This may be because the increased OSG content leads to an increase in film thickness, a closer and more ordered molecular arrangement of OSG, an improvement in material continuity and density, and a corresponding increase in stiffness, which is conducive to an increase in TS [39]. The WVP initially decreased and then increased. This could be attributed to the increase in OSG concentration, leading to an increase in the number of hydrophilic groups and creating a denser matrix network structure. This densification enhanced the barrier effect against water vapor [10,32]. However, when the added amount of OSG exceeded 17.5 mg/mL, the WVP value of the composite film increased. This might be due to an increase in OSG, causing an overexposure of hydrophilic groups in the film-forming solution. These groups more readily interacted with water molecules in food or air, thus increasing the WVP of the composite film.

Figure 1B shows that the TS of the composite membrane decreased from 15.64 MPa to 5.46 MPa as the glycerol content increased. By contrast, the EAB of the composite film increased from 31.94% to 70.92% as the glycerol content increased. This may be because as the glycerol content increases, glycerol molecules form new hydrogen bonds with the respective hydroxyl groups on the oxidized starch chains [10,40]. This thus weakens the stronger hydrogen bond interaction between the oxidized starch chains, thereby declining the TS of the film. At this time, the strength of the composite film decreased and exhibited better flexibility. The WVP of the composite film gradually decreased. This reduction could be attributed to glycerol, which is a highly hydrophilic plasticizer. An excessive amount of glycerol led to a restructuring of the network structure of the composite film. This resulted in an increase in free volume, a loosening of the structure, and enhanced molecular fluidity. Therefore, glycerol could more easily absorb water molecules from the environment [41].

Figure 1C shows that as the CMC content increased, both the TS and EAB of the composite film increased. Because CMC is also a linear long-chain molecule, the number of linear structures in the composite film per unit volume increases with an increase in its content. CMC increases the thickness of the composite film, thereby leading to an increase in its TS [11,14]. In addition, the fluidity between molecular chains is enhanced by the intertwining between CMC molecules and OSG molecular chains, inducing increased flexibility and EAB of the composite film. With the increase in the CMC content, the groups within the molecule interacted with other substances through hydrogen bonding, van der Waals forces, and other intermolecular forces, forming a tight network structure. This strengthened the overall intermolecular forces in the whole system and enhanced the tightness of the network structure. Therefore, the WVP of the composite film gradually decreased [30].

### 3.2. Analysis of Box–Behnken Response Surface Optimization Results

The data were subjected to variance analysis and twice to multiple regression fitting using Design Expert 13. The regression equation was obtained as follows: Y = 0.562 + 0.1012A − 0.11B + 0.0588C − 0.0875AB + 0.015AC + 0.1275BC − 0.221A^2^ − 0.0135B^2^ − 0.016C^2^.

The variance analysis results are presented in Table 1 and Table 2. The results indicated that the three factors examined in the preparation process of the OSG composite film had extremely significant effects on the comprehensive score (*p* < 0.01). In Table 3, R^2^ = 0.9954, R^2^adj = 0.9896, and CV% = 4.13, indicating that the model had a good fit, strong correlation, and minimal experimental error. A *p* value of 0.1404 > 0.05 indicated that the lack of fit was not significant, and its impact on the test results was negligible. Therefore, the model could be effectively used for analysis and prediction. The findings suggested that the main order of influence of each factor on the comprehensive score was as follows: glycerin content > OSG content > CMC content.

**Figure 2:**
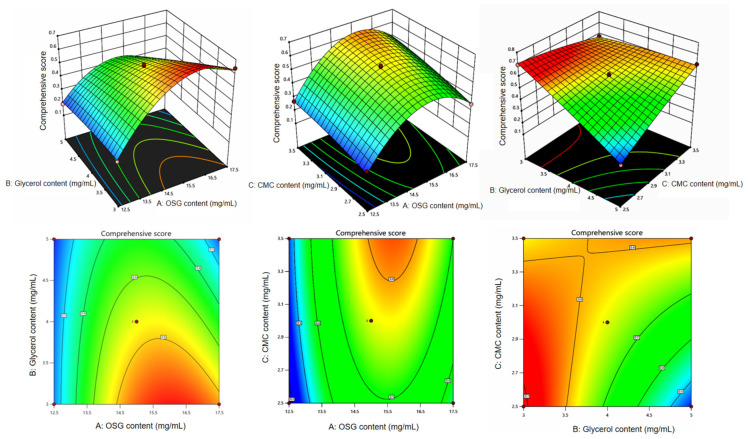
Influence of interaction of various factors on comprehensive score.

The Design Expert 13 software was used to illustrate the interaction among various factors impacting the comprehensive score of the response surface and contour plot (Figure 2). The steeper the slope of the response surface, the greater the influence of test factors on the response value. The more the contour lines tended to form an ellipse, the stronger the interaction between the factors. Conversely, the more the contour lines tended to form a circle, the weaker the interaction between the factors. The optimal extraction process determined via software fitting was as follows: OSG supplemental level at 16.17 mg/mL, glycerol supplemental level at 3.02 mg/mL, CMC supplemental level at 2.8 mg/mL, and the predicted value of comprehensive score at 0.717.

### 3.3. Determination of Antibacterial Activity

**Figure 3:**
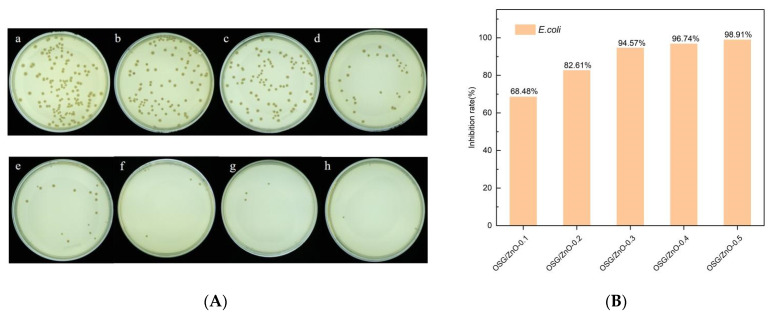
Antibacterial effect of SG/CMC film, SG/CMC film, nano-ZnO composite film with different contents on *Escherichia coli* (**A**,**B**). (**a**) Blank; (**b**) SG/CMC film; (**c**) OSG/CMC film; (**d**) OSG/ZnO-0.1 film; (**e**) OSG/ZnO-0.2 film; (**f**) OSG/ZnO-0.3 film; (**g**) OSG/ZnO-0.4 film; (**h**) OSG/ZnO-0.5 film.

**Figure 4:**
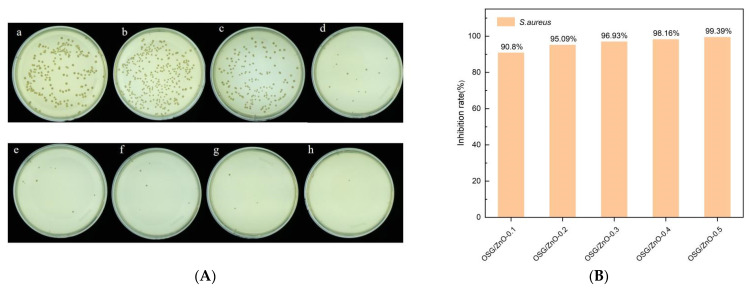
Antibacterial effect of SG/CMC film, SG/CMC film, nano-ZnO composite film with different contents on *Staphylococcus aureus* (**A**,**B**). (**a**) Blank; (**b**) SG/CMC film; (**c**) OSG/CMC film; (**d**) OSG/ZnO-0.1 film; (**e**) OSG/ZnO-0.2 film; (**f**) OSG/ZnO-0.3 film; (**g**) OSG/ZnO-0.4 film; (**h**) OSG/ZnO-0.5 film.

To further improve the application potential of the composite film, the composite film must have antibacterial properties under the premise of better mechanical properties [18]. Nano-ZnO exerts a good antibacterial effect for a long duration [42]. The nano-ZnO antibacterial composite film was prepared by adding nano-ZnO to the OSG-CMC substrate. Figure 3 and Figure 4 present the bacteriostatic effect of nano-ZnO composite films. Single SG and OSG composite films do not display obvious bacteriostatic activity under experimental conditions. When the nano-ZnO content is increased as the main antibacterial agent, more nanoparticles come in direct contact with bacteria, leading to a higher antibacterial activity. When 0.1 mg/mL of nano-ZnO was added, the antibacterial rate against *S. aureus* reached 90.8%, and when the amount used was 0.3 mg/mL, the antibacterial rate against *E. coli* was 94.57%. In summary, the OSG/ZnO composite film exhibited a good inhibitory effect against both *E. coli* and *S. aureus*, and the inhibitory effect on *S. aureus* was greater than that on *E. coli*. The higher inhibition rate of the composite film against *S. aureus* compared with *E. coli* might be attributed to the presence of lipopolysaccharide, phospholipid bilayer, and lipoprotein in the outer membrane of E. coli. These components effectively safeguarded the internal structure of the film from destruction.

### 3.4. Effect Test of the Composite Film on the Wampee Fruit Preservation Period

The antibacterial experiment results revealed that when the amount of nano-ZnO was greater than 0.3 mg/mL, the rates of *E. coli* and *S. aureus* inhibition were greater than 90%. Considering the optical properties, mechanical properties, and moisture resistance of the composite film, a film with a nano-ZnO content of 0.3 mg/mL was selected for the wampee fruit preservation experiment. The TS of the OSG/ZnO-0.3 film was 19.25 MPa, the EAB was 31.03%, the water vapor transmittance (WVP) was 0.745 × 10^−10^ g pa^−1^ s^−1^ m^−1^, and the light transmittance was 40.55%.

**Figure 5:**
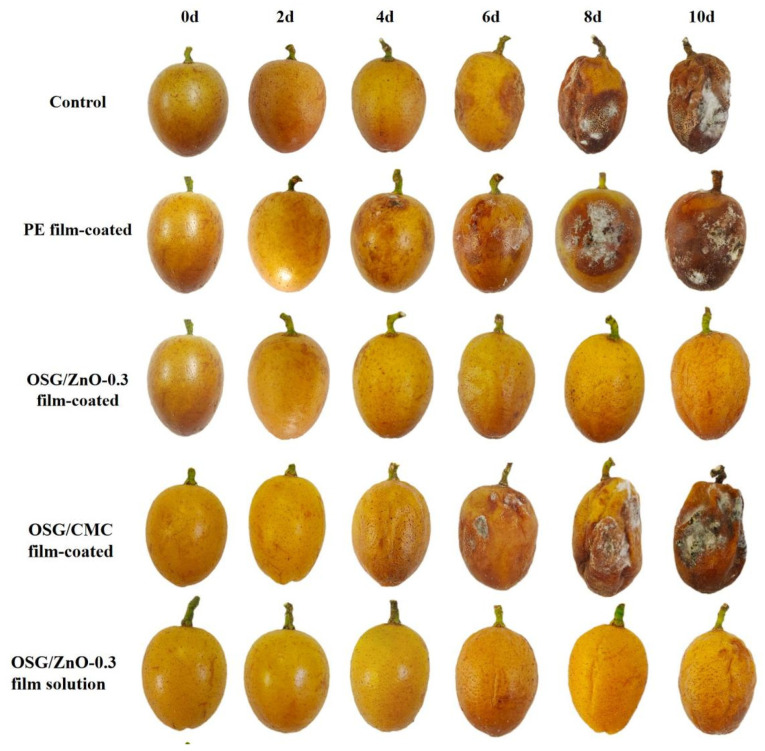
Influence of different preservation methods on the preservation effect of wampee fruit.

Figure 5 demonstrates that noticeable shrinkage and mildew were observed in both the untreated group and the OSG/CMC film-coated group by the 6th day, with complete decomposition occurring by the 10th day. In the PE film-coated group, although severe shrinkage was absent, rotting began on the 4th day. The OSG/ZnO-0.3 film-coated group and coating with the OSG/ZnO-0.3 film solution group only showed the shrinkage phenomenon on the 10th day, with no evidence of rotting, indicating superior OSG/ZnO-0.3 film-coated effectiveness. This underscores the utility of OSG/ZnO for preserving wampee fruit and significantly extending the wampee fruit preservation duration.

#### 3.4.1. Weight Loss Rate

**Figure 6:**
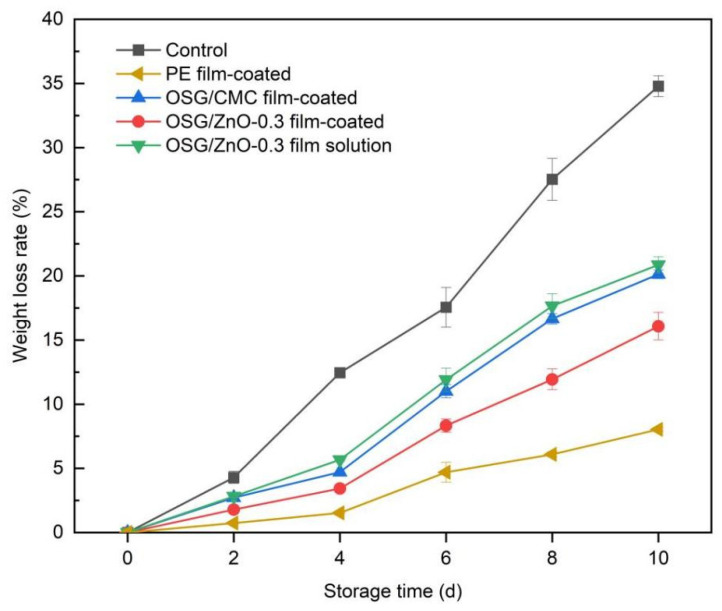
Changes in weight loss rate of wampee fruits during storage.

A primary cause of weight loss of fruits and vegetables during storage is the transfer of water from the interior to the surface and its volatilization in the air [3]. Figure 6 presents the changes in wampee fruit weight during storage. In all experiments, the weight loss rate of wampee fruit exhibited a gradually increasing trend. As the storage time was extended, the weight loss rate of untreated wampee fruit became gradually higher than that of fruits from other treatment groups, followed by those of the group of wampee fruit that was immersed in the OSG/ZnO-0.3 film solution and directly exposed to room temperature. Because the blank group was directly exposed to air without any treatment, respiratory and transpiration effects were relatively high, nutrients were rapidly consumed, water loss was intensified, and fruits wrinkled and lost their edible value [30]. After the wampee fruit was soaked in the film liquid, the film formed after drying had a certain barrier property, but the film was too thin, which also led to a high loss in weight. The weight loss was lower in the OSG/ZnO-0.3 film-coated group than in the OSG/CMC film-coated group. This is because the composite film had a lower WVP value and a certain gas barrier property, and the barrier property improved after nano-ZnO was added. This barrier property could better reduce the respiration and transpiration of wampee fruit and reduce the loss of water and nutrients.

In addition, the weight loss rate of the PE group was the lowest, mainly because the WVP value of the PE cling film was low. Moreover, the water vapor generated through wampee fruit respiration and transpiration condensed on the fruit surface, resulting in low water loss. However, as the condensation of water on the wampee fruit surface provided a favorable environment for microbial growth (Figure 5), the PE group fruits first began to mold and rot.

#### 3.4.2. Rate of Rotting

**Figure 7:**
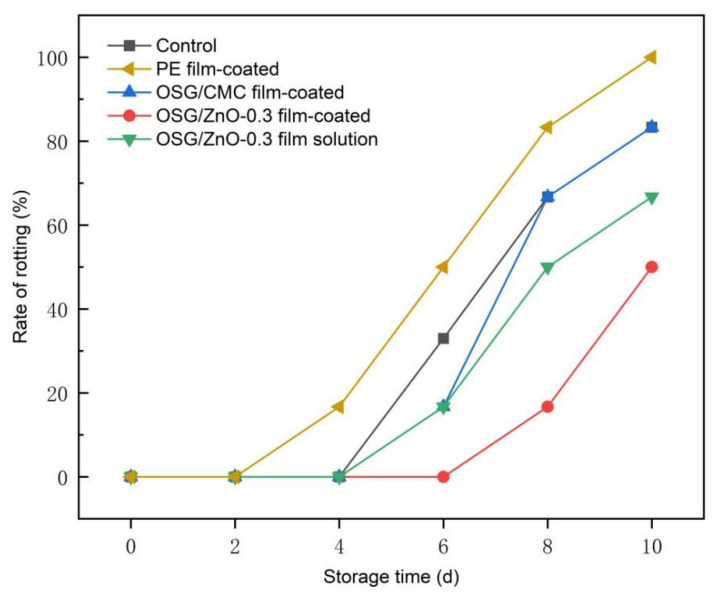
Changes in rate of rotting of wampee fruits during storage.

Wampee fruit is a thin and juicy fruit. These fruits easily lose water during postharvest storage, causing the skin to dry, brown, and rot [21]. Wampee fruits appeared full and shiny in their initial stage (Figure 5). As the storage time was extended, the surface luster of the fruit gradually disappeared, and shrinkage and rotting occurred. The wampee fruits in the PE group began to rot on the 4th day of storage, and the rotting rate reached 50% on the 6th day (Figure 7). This was mainly because the WVP value of the PE cling film was very low, which led to the condensation of wampee-evaporated water on the fruit surface, thereby providing a favorable environment for microbial growth. The blank group and the OSG/ZnO film-coated group exhibited extensive rotting from day 6. By contrast, the OSG/ZnO-0.3 film-coated group and coating with the OSG/ZnO-0.3 film solution group exhibited good anti-corrosion effects, especially with the OSG/ZnO-0.3 film-coated group. Because the film solution coated on wampee fruit was directly exposed to air, the film formed on the surface was relatively thin and could not serve as a good barrier. This also proves that the OSG and nano-ZnO combination can effectively maintain the cell structure of wampee fruit, delay fruit rot, and make the fruits less susceptible to rotting [1].

#### 3.4.3. Total Soluble Solid Content

**Figure 8:**
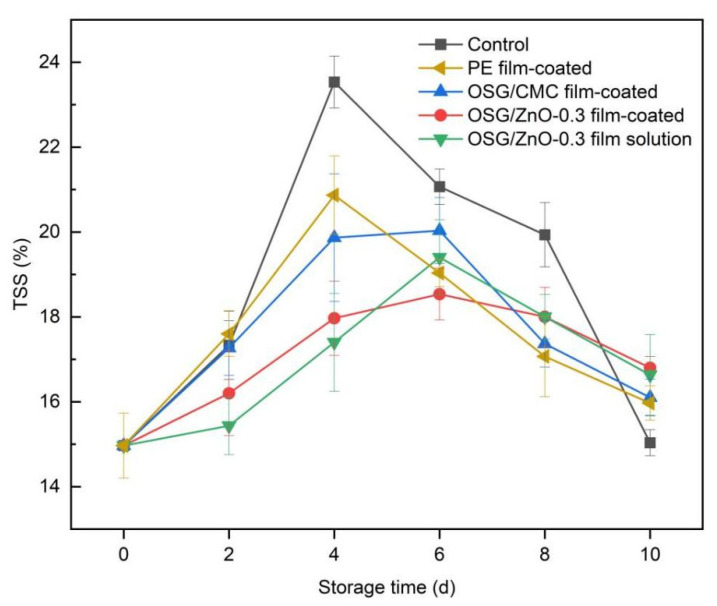
Changes in soluble solid content (TSS) of wampee fruits during storage.

TSS is formed by water-soluble compounds of substances such as sugars, acids, vitamin C, and some pectin. TSS can indicate the total sugar content in the fruit and the fruit ripening state [41,43]. The TSS content increased first and then decreased (Figure 8) because the content of sugar and other soluble substances increased as fruit maturity increased during storage. A trend of decrease in the TSS content was noted because the fruit begins to age at the late storage stage and relies on the decomposition of its nutrients to maintain respiration, resulting in TSS depletion. Compared with the control group, the change trend of TSS content in fruits coated with the film solution and the OSG/ZnO-0.3 film-coated fruit was smaller because it had higher antibacterial properties. This property allowed the effective inhibition of microbial growth, thereby preventing the wampee fruit from being infected by mold during storage. Meanwhile, the barrier property of the composite film allowed the formation of a low O_2_ atmosphere and inhibited fruit respiration. Thus, the consumption of soluble solids was reduced and the change in the TSS content was delayed. The TSS content of the PE group decreased rapidly after the 4th day because part of the wampee fruit in this group began to rot from the 4th day, and fruit nutrients were rapidly consumed by microorganisms.

#### 3.4.4. Titratable Acidity

**Figure 9:**
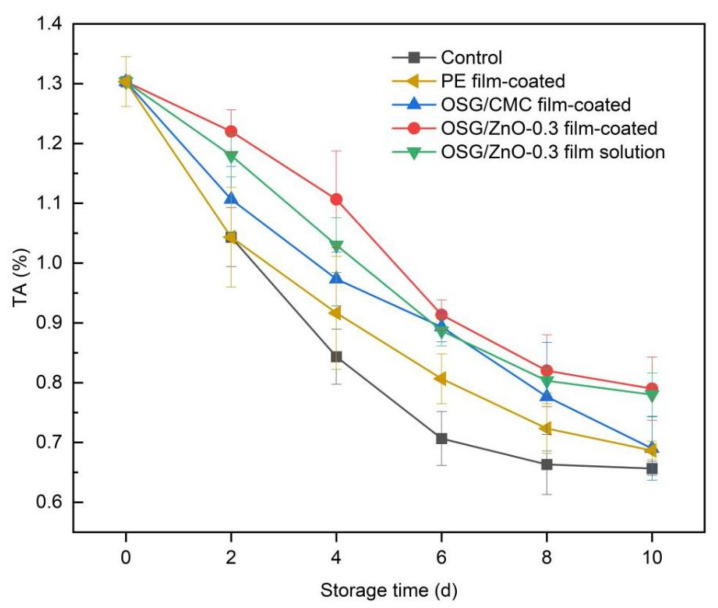
Changes in titrable acidity (TA) of wampee fruits during storage.

The TA is a component that plays a crucial role in maintaining fruit quality and fruit flavor quality [28]. As shown in Figure 9, when the storage time is extended, the organic acid content of fruits gradually decreases, and TA in all groups exhibited a trend of continuous decline. When organic acids are gradually consumed as substrates during plant respiration, titratable acidity decreases during storage. The influence of different film packaging materials on titratable acidity is also obvious. The TA content in the control group decreased the most, and both the coating with film solution and coating with film decelerated the reduction rate of TA content in wampee fruit (Figure 9). The OSG/ZnO-0.3 film-coated allows sufficient gas permeability and water vapor transmission, thereby reducing the oxygen content in the membrane microenvironment. This thus delays the fruit respiration rate during storage and reduces TA consumption. Moreover, the OSG/ZnO-0.3 film-coated effectively inhibited the invasion, growth, and reproduction of microorganisms in the external environment and reduced the rate of oxidative decomposition of wampee tissues, thereby decelerating the total acid consumption.

## 4. Conclusions

Nowadays, reports on SG film formation are scarce. No research has explored the preparation of modified SG composite film and its application in fruit preservation. In this study, OSG was prepared via oxidation modification of SG. Nano-ZnO was used as an antibacterial agent, mixed with CMC and glycerol to prepare antibacterial composite film and explore its antibacterial activity. When 0.3 mg/mL of nano-ZnO was used, the rates of *E. coli* and *S. aureus* inhibition were more than 90%, and the physicochemical properties of the film were better. The effective use of the OSG/ZnO-0.3 composite film for preserving wampee fruit is demonstrated by the TSS and TA content decline, along with the extended storage duration. In general, the OSG/CMC composite film, incorporating nano-ZnO, exhibits robust mechanical properties and antibacterial activity, rendering it a viable material for fresh-keeping fruit packaging. This material holds significant promise for broader applications in active food packaging, offering a novel avenue for SG utilization and new food packaging materials. The current investigation focuses solely on the fresh-keeping effects for fruits, with ongoing research aimed at assessing its effectiveness in preserving vegetables.

## Figures and Tables

**Table 1:** Response surface methodology results for optimization of the preparation process of OSG composite film.

Run	OSG Content	Glycerol Content	CMC Content	Comprehensive Score
	(A)/mg mL^−1^	(B)/mg mL^−1^	(A)/mg mL^−1^	
1	12.5	3	3	0.24
2	17.5	5	3	0.24
3	15	5	2.5	0.23
4	15	4	3	0.58
5	17.5	4	2.5	0.35
6	12.5	4	2.5	0.2
7	15	5	3.5	0.62
8	15	4	3	0.57
9	12.5	5	3	0.19
10	15	3	2.5	0.7
11	15	4	3	0.55
12	12.5	4	3.5	0.27
13	15	4	3	0.55
14	17.5	4	3.5	0.48
15	17.5	3	3	0.64
16	15	3	3.5	0.58
17	15	4	3	0.56

**Table 2:** Analysis of variance of OSG composite film regression model.

Source	Sum of Squares	df	Mean Square	F-Value	*p*-Value	
Model	0.5149	9	0.0572	170.04	<0.0001	significant
A-OSG content	0.082	1	0.082	243.77	<0.0001	**
B-Glycerol content	0.0968	1	0.0968	287.73	<0.0001	**
C-CMC content	0.0276	1	0.0276	82.08	<0.0001	**
AB	0.0306	1	0.0306	91.03	<0.0001	**
AC	0.0009	1	0.0009	2.68	0.1459	
BC	0.065	1	0.065	193.28	<0.0001	**
A^2^	0.2056	1	0.2056	611.26	<0.0001	**
B^2^	0.0008	1	0.0008	2.28	0.1747	
C^2^	0.0011	1	0.0011	3.2	0.1166	
Residual	0.0024	7	0.0003			
Lack of Fit	0.0017	3	0.0006	3.28	0.1404	not significant
Pure Error	0.0007	4	0.0002			
Cor Total	0.5172	16				

Note: ** means the difference is very significant (*p* < 0.01).

**Table 3:** Model simulation analysis.

Analysis	Data	Analysis	Data
Std. Dev.	0.01834	R^2^	0.9954
Mean	0.4441	Adjusted R^2^	0.9896
C.V.%	4.13	Predicted R^2^	0.9461
		Adeq Precision	37.7639

## Data Availability

The original contributions presented in the study are included in the article, further inquiries can be directed to the corresponding author.
